# The frequency of potentially inappropriate medication usage in community-dwelling oldest-old people

**DOI:** 10.55730/1300-0144.5835

**Published:** 2024-04-04

**Authors:** Fuat Nihat ÖZAYDIN

**Affiliations:** Department of Pharmacology, Faculty of Medicine, İstanbul Atlas University, İstanbul, Turkiye

**Keywords:** Aged, potentially inappropriate medication list, polypharmacy

## Abstract

**Background/aim:**

It is critically important to protect the health of the oldest-old people, as their hospitalization and death rates are high. The objective of this study was to analyze the prevalence of potentially inappropriate medication use among the community-dwelling oldest-old people and its association with their demographic characteristics.

**Materials and methods:**

Data were collected from real-world settings using the observational method for this descriptive study. An older adult aged ≥ 85 years old was defined as the oldest-old. The participants were visited in their homes. The generic names of the medications used, and the age, sex, and province of residence were recorded. The medications were analyzed according to the 2019 Beers criteria, and their prevalence of use among the oldest-old people was determined.

**Results:**

Data were collected from 549 of the oldest-old people. The median age of the participants was 88.0 years (88.8 ± 3.5; min = 85.0, max = 102), and 61.3% (n = 336) of them were female. The study findings showed that 65.0% of the community-dwelling oldest-old people used potentially inappropriate medications, with a median number of 1 (min = 0, max = 6). The prevalence of potentially inappropriate medication use increased linearly with the number of drugs used (p = 0.001). The median number of medications was significantly higher in the potentially inappropriate medication user group (5 vs. 2, p = 0.001). Diuretics, proton pump inhibitors, and nonsteroidal antiinflammatory drugs were the most frequently used potentially inappropriate medications.

**Conclusion:**

The prevalence of potentially inappropriate medication use was high among the oldest-old people in Turkiye. There were no differences in frequency of use according to age, sex, or geographical region. It is important to prevent the use of potentially inappropriate medications that should be avoided and to monitor the oldest-old group that uses potentially inappropriate medications that should be used with caution.

## 1. Introduction

Old age is classified as youngest-old (65–74 years old), middle-old (75–84 years old), and oldest-old (≥85 years old) [1]. Similarly, the Turkish Statistical Institute defines old age as 65 years and above, and divides old age into three groups: 65–74, 75–84, and ≥85 years. The number of the oldest-old people was 667,681, and their rate in the older adult group was 7.9% in Turkiye. The growth rate of the oldest-old people has remained lower than that of older adults^1^. The healthy life expectancy in the oldest-old population was 1.4 years in 2014–2016, and decreased to 1.1 years in 2017–2019^2^. These findings highlight the critical importance of disease prevention, and, by all necessity, there ought to be rational drug use for treatments, especially among the oldest-old.

Guides have been published indicating medications called potentially inappropriate medications (PIMs) that may be associated with adverse reactions, hospitalization, and death among the older adults [2]. Although hospitalization, length of hospital stay, and mortality rates are higher among the oldest-old people, there are no specific guides for PIMs. There are guides in place for the older people. Among the guides developed according to explicit criteria, the Beers criteria and Turkish Inappropriate Medication use in the Elderly (TIME) criteria, which is specific to Turkiye, are some of those [3,4]. Beers criteria are typically lists of medications or criteria that can be applied with little or no clinical judgment and do not account for individual differences among patients [3].

Except for certain groups, such as antibiotics and benzodiazepines, medications without a prescription can be purchased from pharmacies for self-rescue in Turkiye. Approximately 45% of medications in the PIM group are available over the counter from pharmacies in Turkiye [5]. These nonprescribed medications are not recorded in the health records of the oldest-old people. For all of these reasons, it is necessary to collect data from a real-world setting via observational methods to identify all medications used by the oldest-old people [6]. Collecting data through observational methods in a real-world setting is obviously possible with the use of guides prepared according to explicit criteria.

The objective of this observational study was to perform a PIM analysis of the medications used in the community-dwelling oldest-old group according to the 2019 Beers criteria and their association with demographic characteristics.

## 2. Materials and methods

### 2.1. Design and setting

This research was a descriptive, cross-sectional study. Data were collected between December 2021 and May 2022. Students enrolled in a medical undergraduate program at a university and taking a pharmacology course were selected as interviewers. After a course on the basic principles of pharmacology (introduction to pharmacology, pharmaceutical forms of medications, and routes of administration), 120 students agreed to participate in the study as interviewers. The minimum sample size was calculated as 384 at a 95% confidence level, with a 5% margin error and 50% prevalence while applying the sample size formula for a proportion or descriptive study of the Open EpiInfo program^3^. The snowball sampling method was used due to the low number of oldest-old people in Turkiye and their distribution throughout the country [6,7].

The oldest-old group was divided into four age groups: 85–89 years old, 90–94 years old, 95–99 years old, and 100–104 years old.

The provinces where they live were divided into five regions of Turkiye, as follows:(1) West, including Aydın, Balıkesir, Bursa, Çanakkale, Denizli, İstanbul, İzmir, Kırklareli, Kocaeli, Manisa, Muğla, Sakarya, Tekirdağ, and Yalova (Marmara and Aegean); (2) South, including Antalya, Adana, Burdur, Hatay, Isparta, İçel, K.Maraş, and Osmaniye (Mediterranean); (3) Central, including Afyon, Amasya, Ankara, Bilecik, Bolu, Çankırı, Çorum, Eskişehir, Kayseri, Kırşehir, Konya, Kütahya, Nevşehir, Niğde, Sivas, Tokat, Uşak, Yozgat, Aksaray, Karaman, Kırıkkale, and Düzce (Central Anatolia); (4) North, including Artvin, Giresun, Gümüşhane, Kastamonu, Ordu, Rize, Samsun, Sinop, Trabzon, Zonguldak, Bartın, and Karabük (Black Sea); and (5) East, including Adıyaman, Ağrı, Bingöl, Bitlis, Diyarbakır, Elazığ, Erzincan, Erzurum, Gaziantep, Hakkari, Kars, Malatya, Mardin, Muş, Siirt, Tunceli, Şanlıurfa, Van, Bayburt, Batman, Şırnak, Ardahan, Iğdır, and Kilis (Eastern and Southeastern Anatolia)^4^.

### 2.2. Methodology

Whenever an oldest-old adult used two or more medications, it was defined as polypharmacy [8]. Polypharmacy was divided into three categories: minor (2–4 medications), major (5–9 medications), and hyper: (≥10 medications) [9]. The 2019 Beers criteria were used for the PIM analysis [3].

The research was conducted throughout Turkiye. The oldest-old neighbors and relatives were visited by interviewers in their homes, and face-to-face interviews were conducted. The age, sex, and province of residence were recorded. The generic names of oral, parenteral, and inhaled medications were recorded for the study. The oldest-old people staying in inpatient institutions, such as hospitals and nursing homes, were not included in the study.

Ethical approval was granted by the Istanbul Okan University Ethics Committee (08.09.2021/141). This study was performed in line with the principles of the Declaration of Helsinki.

### 2.3. Statistical analysis

The data were analyzed using the IBM SPSS Statistics 19.0 package program (IBM Corp., Armonk, Chicago, IL, USA). Categorical variables were presented as numbers and percentages. The Kolmogorov–Smirnov test was performed as a normality test. Nonparametric tests were used because the continuous data were not normally distributed. Nonparametric distributed continuous variables were presented as median, minimum, and maximum values. Mann–Whitney U and Kruskal–Wallis tests were performed for continuous variables, chi-squared tests were performed for categorical variables, and Spearman’s correlation coefficient was calculated. If p < 0.05, the results were considered statistically significant.

## 3. Results

Data were collected from 549 of the oldest-old people. The median age of the participants in the study was 88.0 years (88.8 ± 3.5; min = 85.0, max = 102). The distribution of males and females in age groups was similar (p = 0.341). Moreover, the distribution of men and women across regions was similar (p = 0.826). The sociodemographic characteristics of the participants are shown in Table 1.

Only 2.7% (n = 15) of the oldest-old people were not using medications, 8.5% (n = 47) were using one medication, and 88.8% (n = 487) were using multiple medications. In the polypharmacy group, 57.8% (n = 281), 35.9% (n = 175), and 6.3% (n = 31) were taking 2–4, 5–9, and 10 or more medications, respectively. The median number of medications was 4.0, and the median number of PIMs was 1.0. There was no significant difference in the distribution of the median number of PIMs according to sex, age, and region. However, the median number of PIMs tended to decrease in the 100 and above group compared to the younger age groups. The distribution of the median number of PIMs is summarized in Table 2.

Among the oldest-old people, 65.0% (n = 357) were using one or more PIMs. Of these, 31.5% (n = 173) were using one PIM, 32.3% (n = 177) were using 2–4 PIMs, and 1.2% (n = 7) were using 5–9 PIMs. The frequency of PIM use did not change with sex, age, or region. However, it tended to decrease in the 100 and above group compared to the younger age groups. Distribution of participants using and not using PIMs are shown in Table 3. A positive moderate statistically significant correlation was found between the total number of medications used and the total number of PIMs used (r = 0.571, p = 0.001). The relationship between the total number of medications and the total number of PIMs are shown in Figure. The median number of medications used was significantly lower in the non-PIM user group than in the PIM user group (2 and 5, respectively; p = 0.001).

The PIMs used by the oldest-old people belonged to 42 different medication groups and were available in all classifications (five different classifications) defined in the 2019 Beers criteria. For example, nonsteroidal antiinflammatory drugs (NSAIDs) were in the “PIM use” group, while selective serotonin reuptake inhibitors (SSRIs) and diuretics were in the “use with caution” group, FXa inhibitors were in the “avoid or have their dosage reduced with varying levels of kidney function” group, and nondihydropyridine calcium channel blockers were in the “PIM use due to drug-disease or drug-syndrome” group.

The three most frequently used PIMs by the oldest-old people were proton pump inhibitors (PPIs), NSAIDs, and diuretics. Lansoprazole was used most frequently in the PPI group, and naproxen was used most frequently in the NSAID group. In the diuretic group, hydrochlorothiazide, furosemide, spironolactone, and indapamide were used. In the SSRIs group, escitalopram, sertraline, citalopram, paroxetine, fluoxetine, and vortioxetine were used. In the antipsychotic group, quetiapine, olanzapine, haloperidol, and risperidone were used by the oldest-old people. Rivaroxaban, apixaban, and edoxaban in the FXa inhibitor group, doxazosin and terazosin in the Alpha-1 blocker group, and diltiazem and verapamil in the nondihydropyridine calcium channel blocker group were used. Among the identified PIMs, only ciprofloxacin was included in the infectious disease management group. Top 10 PIMs used in the oldest-old adults including Beers’ classification were presented in Table 4. If the classification was made as the top 10 PIMs used in the group of noncommunicable diseases, gabapentin (n = 7, 1.3%) and corticosteroids (n = 7, 1.3%) were included instead of ciprofloxacin.

The lowest number was in the “drug–drug combinations that should not be used in the older adult group.” Only eight (1.4%) of the oldest-old people were in this group. Drug–drug interactions observed in this study were the combinations of medications with strong anticholinergic effects (paroxetine+olanzapine, paroxetine+solifenacin, and cyproheptadine + chlorpheniramine) and a combination of three or more active medications in the central nervous system, such as antidepressants, antipsychotics, and benzodiazepines (haloperidol+aripip-razole+venlafaxine, haloperidol+quetiapine+escitalopram+alprazolam, haloperidol+escitalopram+alprazolam, halo-peridol+olanzapine+sertraline [two people]).

## 4. Discussion

This study was the first to investigate the frequency of PIM use among the community-dwelling oldest-old people in the real-world setting using an observational method. The frequency of PIM use was high among the community-dwelling oldest-old people throughout Turkiye. The use of PIM was observed for all sexes, age groups, and geographic regions.

The frequency of potentially inappropriate prescribing (PIP) in community-dwelling older adults in Europe was investigated by a review of literature published between 2000 and 2014. Fifty-two manuscripts were examined in this review, and only three studies with an average age of 85 and above were identified [10]. In a study published in 2008, the records of 230,000 older patients registered in the UK Primary Care Patient Record Database were examined [11]. Thirteen percent of the participants were ≥85 years old. Beers 2003 criteria were used to perform PIP analysis. The frequency of PIP was detected in 34.7% of the oldest-old people. The frequency of PIP of antidepressants, sedatives, or anxiolytics was found to be higher in the oldest-old compared to the younger older adults [11]. In a study involving 354 community-dwelling older adults (≥65), the average age was found to be 85.8 ± 4.8, and the frequency of PIM use was 26% according to the 2003 Beers criteria [12]. The only molecule that was similar to our study was doxazosin. In a study that involved 78 community-dwelling oldest-old people (≥85 years), inappropriate prescription (IP) analysis was performed using Beer’s criteria released in 1991 and the “Screening Tool of Older Person’s Prescriptions” (STOPP) released in 2008. The “Screening Tool to Alert to Right Treatment” (START) criteria were not included. Primary health care records of the oldest-old people were examined [13]. In that study, the mean number of medications was 6.1. IPs were detected in 69.2% of the participants. Of these, 34.6% had one IP, and 34.6% had two or more IPs. PIMs were detected in 65.0% of the participants in our study. Of these, 31.5% of the participants had one PIM, and 33.5% had two or more PIMs. The results were thought to be similar. Loop diuretics, SSRIs, and NSAIDs were the PIMs detected in both studies.

In a study conducted with community-dwelling older patients (≥ 65, n = 8235) in China, electronic medical records of the patients were collected, and the frequency of PIM use was investigated using the 2019 Beers criteria [14]. In that study, 12.09% (n = 996) of the participants were ≥85 years old, and the frequency of PIM use in this community-dwelling oldest-old patients was found to be 44.78%. Diclofenac (NSAIDs) and olanzapine (antipsychotic) were the most frequently used PIMs in both studies.

In 2023, a metaanalysis examining the use of PIM among older adults in outpatient services worldwide was published [15]. Benzodiazepines emerged as the most commonly used PIM globally, consistently appearing as the most frequently used in all international studies reviewed [11–14]. However, this result was not observed in the present study. In our study, the diuretic group ranked first, contrasting with the metaanalysis [15] where it was absent and placed second in one study [13]. According to the metaanalysis, the most widely used PIMs worldwide were NSAIDs in second place, PPIs in third place, antidepressants in fourth place, and antipsychotics in fifth place. These molecules are among the PIMs commonly used in studies [11–14]. In our study, PPIs ranked second, NSAIDs ranked third, antidepressants ranked fifth, and antipsychotics ranked sixth. While low-dose aspirin and FactorXa inhibitors were included in our study, they were among the ten most commonly used PIMs as antithrombotic agents in the world metaanalysis. No difference in age-related use of PIM was observed in the world metaanalysis [15]. The findings of our study were consistent with the worldwide prevalence of PIMs, except in the diuretic and benzodiazepine groups. In the metaanalysis, the Beers 2019 criteria were the most sensitive.

In this metaanalysis, it was stated that the frequency of PIM use has increased in the last 20 years toward the present day. There were differences in the prevalence of PIM between geographical regions of the world, and the frequency of PIM use was higher in the ≥80 years old patients [15]. The prevalence of PIM was low (26%–34.7%) in studies conducted before 2010 [11,12]. The frequency of PIM use increased (44.78%) in a later study [14]. Finally, this value was higher in the present study. The results of a study conducted in 2011, in which the prevalence of PIM was high, did not agree with these findings [13]. South America, where the study was conducted, was one of the geographical regions where the use of PIM was the highest in the world [15].

Some studies have been conducted in Turkiye. One included 322 patients aged 65 and above who applied to the home care unit [16]. Twenty-eight of the participants (n = 91) were ≥86 of age and the 2015 Beers criteria were used to analyze PIMs. It was found that 63.7% of the oldest-old group used PIMs in that study. The rate of PIM use was higher among those using more medications. The rate of PIM use was also higher in those using over-the-counter medications. In our study, the rate of PIM use was 65%, with polypharmacy contributing to this increase. The results were evaluated as similar.

In a very recent study conducted in İstanbul, prescriptions of middle- and oldest-old patients (≥80 years old, n = 134,079) diagnosed and treated for essential hypertension by primary care physicians were analyzed. It was determined that 2.4% of the prescribed medications belonged to the PIM group, and 8.8% of them contained at least one PIM in the oldest-old group. NSAIDs and PPIs were identified as the most commonly prescribed PIMs in that study. In our study, these medications were PIMs used at the 2nd and 3rd frequencies. Diuretics (hydrochlorothiazide, furosemide, spironolactone, indapamide), doxazosin, and diltiazem were PIMs belonging to the antihypertensive group in both studies. Similar to the use of ciprofloxacin detected in our study, the nitrofurantoin in that study was determined as PIM belonging to the infectious diseases management group [17]. The PIM analysis was conducted only on the medications used by patients diagnosed with hypertension in that study. No restriction on indication was made in our study.

A study was conducted in Turkiye to investigate the frequency of cardiovascular PIMs used by older adults across the country [18]. Prescriptions registered in the “Prescription Information System” of the Ministry of Health and those written at the primary care level by family physicians in 2015–2016 were analyzed according to Beers 2019 criteria. Cardiovascular PIMs were detected in the prescriptions of 11.56% of participants in all regions of Turkiye. The rate of PIM prescription was higher in patients aged 80 years and older. Doxazosin (α-1 blocker), diclofenac (NSAIDs), and verapamil (nondihydropyridine calcium channel blocker) were the most frequently used PIMs in both studies. The reason for the lower PIM rate in that study compared to our study may be that only the cardiovascular PIM group was analyzed, and PIMs that required dose adjustment or discontinuation according to renal function were not analyzed in that study. In our study, the analysis of all medications used by the oldest-old people was done using all classification of Beers 2019 criteria. PIM use was detected in the oldest-old people living in all geographical regions of Turkiye in both studies. In our study, unlike the compared study, there was no statistical difference in the frequency of PIM use between regions. In the second study, the rate was higher in the Black Sea and Western Anatolia, including İstanbul, compared to other regions of Turkiye.

PIMs were commonly used in all geographical regions of Turkiye [18]. This finding was consistent with those of the present study. The prevalence of PIM use was low in both studies. This is because the methods used in these studies were different [17,18]. In a recently published national study conducted using a method similar to our study, the prevalence of PIM was found to be high, similar to the results of our study [16]. As a result, the prevalence of PIM use was high in the oldest-old group worldwide and in Turkiye, and the types of PIM were similar.

In one study, the use of PIM was examined in the entire older population (n = 431,625) who lived in the Lithuanian region and had mandatory health insurance [19]. Medications registered with the National Health Insurance Fund, affiliated with the Ministry of Health in 2015, were included. The Beers 2019 criteria were applied, and the prevalence of PIM increased with age. However, the prevalence decreased after the age of 85 (65–74: 33.2%; 75–84: 47.8%; 85–94: 18.3%; and ≥95: 0.7%). In addition, it has been reported that the prevalence of PIM use in community-dwelling older people (≥65) increases until the age of 85 and then decreases [20]. However, a study showing the opposite results was also published. A study analyzing 732,228 elderly people (≥75 years) registered in the Swedish Prescribed Drug Register during October–November 2005 found that the odds ratio of inappropriate medication use after 80 increased linearly with age (75–79 age group: 1; 80–84 age group: 1.04; 85–89 age group: 1.13; and ≥90 age group: 1.29). Indicators developed by the National Board of Health and Welfare were used [21]. In our study, PIM use by the oldest-old people decreased only in the later phase (the centennial group). The shortening of the healthy lifespan and the increasing frequency of illness in the oldest-old people may have been the reasons for the increase in the frequency of PIM use until 99 years of age in our study. Only the oldest-old people with better health can use less medication and enter the centenarian group.

This study has some strengths. It was a real-world study using an observational method. It was the first study to analyze the use of PIMs without any kind of restrictions in the oldest-old population in Turkiye and to provide preliminary findings. PIM use analysis was conducted according to sex, age, and region subgroups.

This study also has some limitations. The sample of oldest-old people chosen was not representative of Turkiye in general. Another weakness of the study was the low number of oldest-old people aged 100 years and older. The 2019 Beers criteria were not specifically developed for Turkiye. There would be differences in the medications on the market and the prescribing behavior of physicians in the U.S. and Turkiye. Therefore, its use in Turkiye for PIMs detection might create a handicap with a possibility of overlooking PIM. Diseases existing in the oldest-old people who use medication could not be identified. The laboratory values of the patients could not be obtained. The oldest-old people were not asked about the dosage of their medications for kidney function or the duration of drug use. Prospective follow-up in terms of adverse effects and hospitalization was not conducted. There was no classification of medications as prescription or nonprescription use. The results were based on the statements of the participants. It is possible that incorrect or incomplete answers were given to the questions.

## 5. Conclusion

In our study, the prevalence of PIM use among the community-dwelling oldest-old people across Turkiye was high. There was no significant difference in PIM use when analyzed by sex, age, or geographic region. The pioneering findings of this study would be an important contribution to the development of health policies for the oldest-old people. Further studies with explicit tools, especially with the ones that fit more to the local market and prescribing practice will probably reveal the prevalence and determinants of PIM more accurately.

## Figures and Tables

**Figure:**
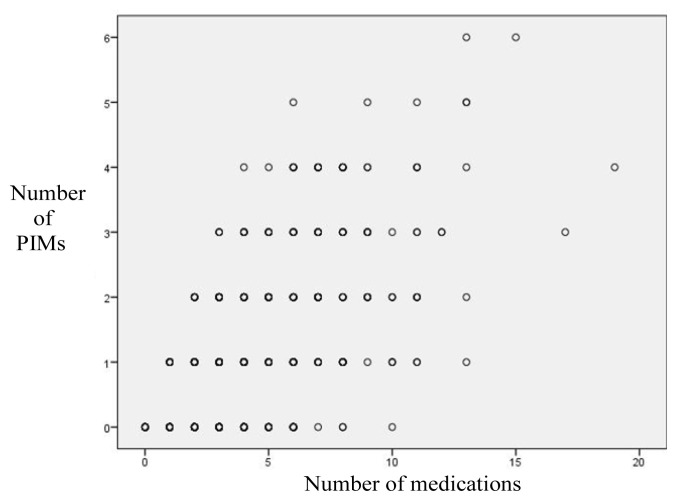
The relationship between the total number of medications used and the total number of PIMs used.

**Table 1:** Sociodemographic characteristics of the participants.

Variable	Sex			p
	Male n (%)	Female n (%)	Total n (%)	
Age groups (years of age)				
85–89	155 (41.1)	216 (58.9)	367 (100)	0.341*
90–94	45(33.0)	91 (67.0)	136 (100)	
95–99	14 (35.0)	26 (65.0)	40 (100)	
100–104	3 (50.0)	3 (50.0)	6 (100)	
Regions				
West	177 (39.0)	276 (61.0)	453 (100)	0.826*
South	5 (33.3)	10 (66.7)	15 (100)	
Central	5 (55.5)	4 (44.5)	9 (100)	
North	14 (36.8)	24 (63.2)	38 (100)	
East	12 (35.2)	22 (64.8)	34 (100)	
Total	213 (38.7)	336 (61.3)	549 (100)	

* chi-squared test

**Table 2:** Distribution of the PIMs used by participants according to sociodemographic variables.

Variable	n (%)	Median (min–max)	p
The average number of per person	549 (100)	1.00 (0–6)	
Sex			
Female	336 (61.3)	1.00 (0–6)	0.431*
Male	213 (38.7)	1.00 (0–5)	
Age groups (years of age)			
85–89	367 (66.9)	1.00 (0–6)	0.558**
90–94	136 (24.8)	1.00 (0–5)	
95–99	40 (7.2)	1.00 (0–5)	
100–104	6 (1.1)	0.00 (0–2)	
Regions			
West	453 (82.5)	1.00 (0–6)	0.301**
South	15 (2.8)	1.00 (0–4)	
Central	9 (1.7)	1.00 (0–4)	
North	38 (6.9)	1.00 (0–6)	
East	34 (6.1)	1.00 (0–4)	

* Mann–Whitney U test,

** Kruskal–Wallis test

**Table 3:** Distribution of participants using and not using PIMs by sociodemographic variables.

Variable	PIMs			p
	(−) n (%)	(+) n (%)	Total n (%)	
Sex				
Male	80 (37.6)	133 (62.4)	213 (100)	0.358*
Female	112 (33.3)	224 (66.7)	336 (100)	
Age groups (years of age)				
85–89	137 (37.3)	230 (62.7)	367 (100)	0.096*
90–94	39 (28.7)	97 (71.3)	136 (100)	
95–99	12 (30.0)	28 (70.0)	40 (100)	
100–104	4 (66.7)	2 (33.3)	6 (100)	
Regions				
West	159 (35.1)	294 (64.9)	453 (100)	0.502*
South	4 (26.7)	11 (73.3)	15 (100)	
Central	1 (11.1)	8 (88.9)	9 (100)	
North	14 (36.8)	24 (63.2)	38 (100)	
East	14 (41.2)	20 (58.8)	34 (100)	
Total	192 (35.0)	357 (65.0)	549 (100)	

* chi-squared test

**Table 4:** Top 10 PIMs used in the oldest-old adults including Beers’ classification.

PIMs	n (%)
Diuretics^1^	122 (22.2)
Proton pump inhibitors^2^	96 (17.5)
Nonsteroid antiinflammatory drugs (NSAIDs)^2^	67 (12.2)
Low dose aspirin^1^	60 (10.9)
Selective serotonin reuptake inhibitors (SSRIs)^1^,^3^,^4^^*^	59 (10.7)
Antipsychotics^1^,^2^,^3^,^4^^**^	58 (10.6)
Factor Xa inhibitors^5^	33 (6.0)
Alpha (α)-1 blockers^2^,^3^	15 (2.7)
Nondihydropyridine calcium channel blockers^3^	12 (2.2)
Ciprofloxacin^5^	10 (1.8)

1 Used with caution in older adults,

2 PIM use in older adults,

3 PIM use in older adults due to drug-disease or drug-syndrome interactions that may exacerbate the disease or syndrome,

4 Drugs with strong anticholinergic properties (^*^for paroxetine) (^**^for olanzapine),

5 Medications that should be avoided or have their dosage reduced with varying levels of kidney function in older adults.

## Data Availability

The data that support the findings of this study are available upon request from the corresponding author.

## Footnotes

1 Türkiye İstatistik Kurumu (2022). İstatistiklerle Yaşlılar, Yaşlı Nüfus 8 milyon 451 bin 669 Kişi Oldu (in Turkish) [online]. Website https://data.tuik.gov.tr/Bulten/Index?p=%C4%B0statistiklerle-Ya%C5%9Fl%C4%B1lar-2022-49667&dil=1#:~:text=Ya%C5%9Fl%C4%B1%20n%C3%BCfus%20olarak%20kabul%20edilen,9%2C9'a%20y%C3%BCkseldi [accessed 15 October 2023].

2 Türkiye Sağlık Enstitüleri Başkanlığı (2021). Türkiye Yaşlı Sağlığı Raporu: Güncel Durum, Sorunlar ve Kısa - Orta Vadeli Çözümler (in Turkish) [online]. Website https://files.tuseb.gov.tr/tuseb/files/yayinlar/20230703124223-FV7IKDhzD1kH-.pdf [accessed 15 October 2023].

3 Open Source Epidemiologic Statistics for Puplic Health (2013). Sample Size for a Proportion or Descriptive Study [online]. Website https://www.openepi.com/SampleSize/SSPropor.htm [accessed 20 March 2021].

4 Hacettepe Üniversitesi Nüfus Etütleri Enstitüsü, Sağlık Bakanlığı Ana Çocuk Sağlığı ve Aile Planlaması Genel Müdürlüğü, Başbakanlık Devlet Planlama Teşkilatı Müsteşarlığı, TÜBİTAK. Hacettepe Üniversitesi Nüfus Etütleri Enstitüsü (2009). Türkiye Nüfus ve Sağlık Araştırması, 2008 (in Turkish) [online]. Website https://fs.hacettepe.edu.tr/hips/dosyalar/Ara%C5%9Ft%C4%B1rmalar%20-%20raporlar/2008%20tnsa/TNSA-2008_ana_Rapor-tr.pdf [accessed 20 March 2021].
